# Narwhal acoustic presence in Eclipse Sound, Nunavut: relationships with sea ice and responses to ships

**DOI:** 10.1038/s41598-025-04032-1

**Published:** 2025-07-02

**Authors:** Jack P. Ewing, Eva Hidalgo-Pla, Alba Solsona-Berga, Kaitlin E. Frasier, Alex J. Ootoowak, Kristin H. Westdal, Sean M. Wiggins, John A. Hildebrand, Joshua M. Jones

**Affiliations:** 1 Scripps Institution of Oceanography, University of California San Diego, La Jolla, 92093 USA; 2 Oceans North, Ottawa, K2P 1R2 Canada

**Keywords:** Environmental impact, Ocean sciences, Behavioural ecology, Biooceanography, Climate-change ecology, Conservation biology, Ecological modelling, Behavioural ecology, Biooceanography, Conservation biology, Ecological modelling

## Abstract

The Arctic Ocean is undergoing rapid sea ice loss and increasing ship traffic, introducing potential stressors for wildlife and challenges for management and conservation. This study examines narwhal (*Monodon monoceros*) responses to vessels in eastern Eclipse Sound, Nunavut, Canada using underwater acoustic recordings and ship tracking data collected between 2016 and 2021. The effect of ship proximity on detection of narwhal echolocation clicks was analyzed, accounting for environmental and temporal factors affecting detection probability. Narwhal acoustic presence exhibits seasonality, peaking in July and October, and is correlated with low solar angle in both seasons and sea ice concentration during ice formation in October. Our analysis revealed an inverse relationship between ship proximity and narwhal acoustic presence in July and October, most pronounced when ships were within 20 km of the recorder in October. These distances suggest that narwhals react to broadband sound pressure levels well below 120 dB re: 1 µPa and are more sensitive to low-frequency sounds (< 1 kHz) than previously assumed. This study offers region- and population-specific insights into narwhal responses to ships, highlighting the importance of integrating long-term monitoring of wildlife, environmental conditions, and human activities to improve prediction of Arctic marine species’ movements and behavior.

## Introduction

Narwhals (*Monodon monoceros*) are endemic to the Arctic, where their seasonal movements are closely tied to the annual advance and retreat of sea ice^1^. However, declining summer sea ice extent and extended ice-free periods across the Arctic may alter the timing of narwhal migrations and the seasonal distribution of their populations^2^. Concurrently, narwhals are exposed to underwater noise from growing shipping traffic, particularly in their habitat areas where tourism and shipping occur during open water periods^1–3^.

Inuit residents of Arctic coastal communities have a profound knowledge of narwhal behavior and have raised concern about the impacts of increasing commercial shipping, particularly underwater noise, on narwhals^2,4^. Their observations are supported by several studies suggesting that narwhals are more sensitive to disturbance from underwater noise than other odontocete species outside the Arctic^5–9^. To inform effective species management and marine spatial planning, there is a need to develop a region-specific understanding of narwhal seasonal interactions with habitat factors such as sea ice, and their responses to anthropogenic stressors such as shipping activity.

**Fig. 1:**
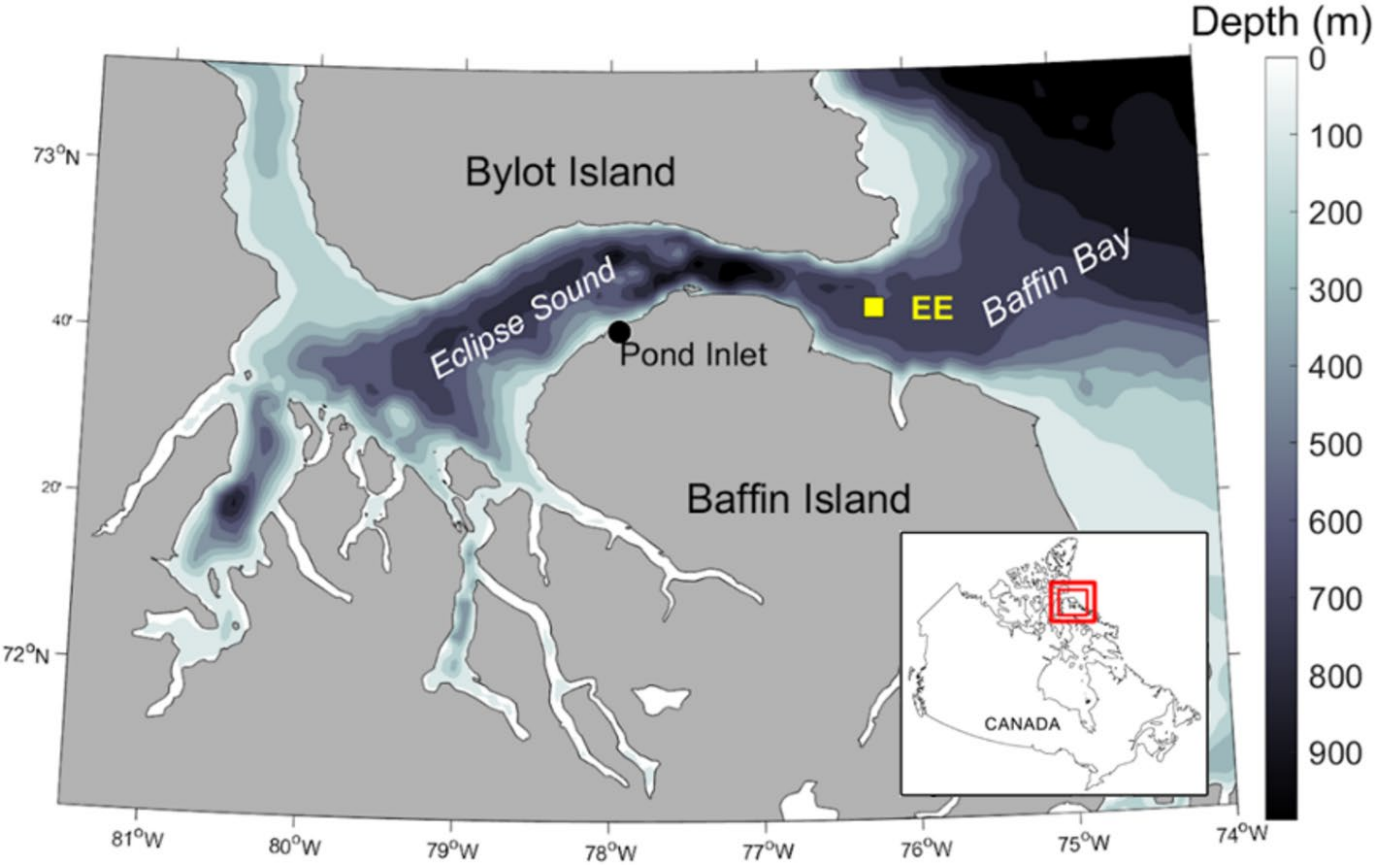
Map of the study area along with inset map for location within the Canadian Arctic (red square). Gray bar represents bathymetric depth in meters. The acoustic recording location (EE, yellow square) is at the eastern entrance to Eclipse Sound in northern Baffin Island, Nunavut, Canada.

Eclipse Sound in the eastern Canadian Arctic (Fig. 1) is a summering area for a distinct population of narwhals^10–14^ and has recently seen a substantial increase in ship traffic, due primarily to the expansion of a regional mining project and an increase in vessel-based tourism^15,16^. This connected system of inlets and fjords is traversed by a shipping corridor used to transport iron ore and other cargo during the open water season^16^. Pond Inlet is a regular stopover for passenger ships and pleasure craft visiting the region. From 2015 to 2019, there was a 384% increase in one-way vessel transits through the eastern entrance to Eclipse Sound, with > 80% of that increase related to the development of a regional mining project and the remaining 20% primarily due to an increase in vessel-based tourism^16^. Summer estimates of narwhal abundance within Eclipse Sound dropped from 20,200 in 2004^13^ to 10,489 in 2013^17^, to 4,381 and 2,081 in 2020 and 2021 respectively^2^. This concurrent decline in Eclipse Sound narwhal regional abundance has elevated concerns about potential shipping impacts on this population.

Previous studies in Eclipse Sound have documented narwhal behavioral responses to ship traffic, including altered vocalization patterns^18^ and altered movement behaviors^3^ at distances up to 10 km from passing ships. Narwhals have also exhibited strong avoidance of areas within 1 km from the bow and stern of passing ships^3^. These studies of Eclipse Sound narwhals focused on the short-term responses of individual animals and were restricted to distances of up to 10 km from ships. There has been limited research on narwhal responses to ships at greater distances and no previous study integrating behavioral responses to ships with potentially confounding influences of temporal and environmental factors, such as time of year and sea ice concentration.

The primary objective of this study is to investigate how narwhal presence at a fixed location (Fig. 1, ‘EE’) within a known summering area is influenced by three categories of factors: temporal (e.g., day of year), environmental (e.g., sea ice concentration), and anthropogenic (e.g., ship proximity). The study is organized into two phases: first, we quantify the temporal variability in narwhal acoustic occurrence in relation to time of year and environmental factors. In the second phase, we assess the influence of ship traffic on narwhal acoustic presence during the months of July and October, two months when both vessels and narwhals are present. Using a multi-year passive acoustic monitoring (PAM) dataset recorded at a fixed location at the eastern entrance to Eclipse Sound (Fig. 1, ‘EE’), satellite Automatic Identification System (AIS) ship location information, and remotely sensed environmental data, we assess narwhal responses to these factors at two temporal scales. The analysis was conducted at an annual time scale with respect to day of year and environmental variables, and then at a timescale of minutes during two peak annual periods of acoustic presence with respect to environmental factors and varying proximity to ships. PAM allows for continuous, non-invasive data collection in remote regions, enabling the detection of narwhal echolocation clicks and the measurement of underwater noise levels associated with passing ships^16^. Narwhal presence within 5.3 km of the recording location is inferred from acoustic detections of their characteristic echolocation clicks. Ship proximity is determined from AIS data transmitted by each ship. By statistically isolating the effects of ship proximity from natural environmental factors, we aim to quantify the relative influence of each variable on narwhal acoustic presence and behavior.

This study reveals a clear annual pattern of narwhal presence at the eastern entrance to Eclipse Sound during periods of sea ice retreat (May through July) and advance (October through November), aligning with the knowledge of Inuit in the region. Within these seasons, narwhal acoustic detection probability is reduced in response to approaching ships at distances of 20 km or more between the ship and recording location. These findings provide valuable insight for management and marine spatial planning in the Eclipse Sound region, quantifying relationships between narwhals and their habitat while observing behavioral sensitivity to ship traffic.

## Results

### Annual presence of narwhals in eastern Eclipse Sound

**Fig. 2:**
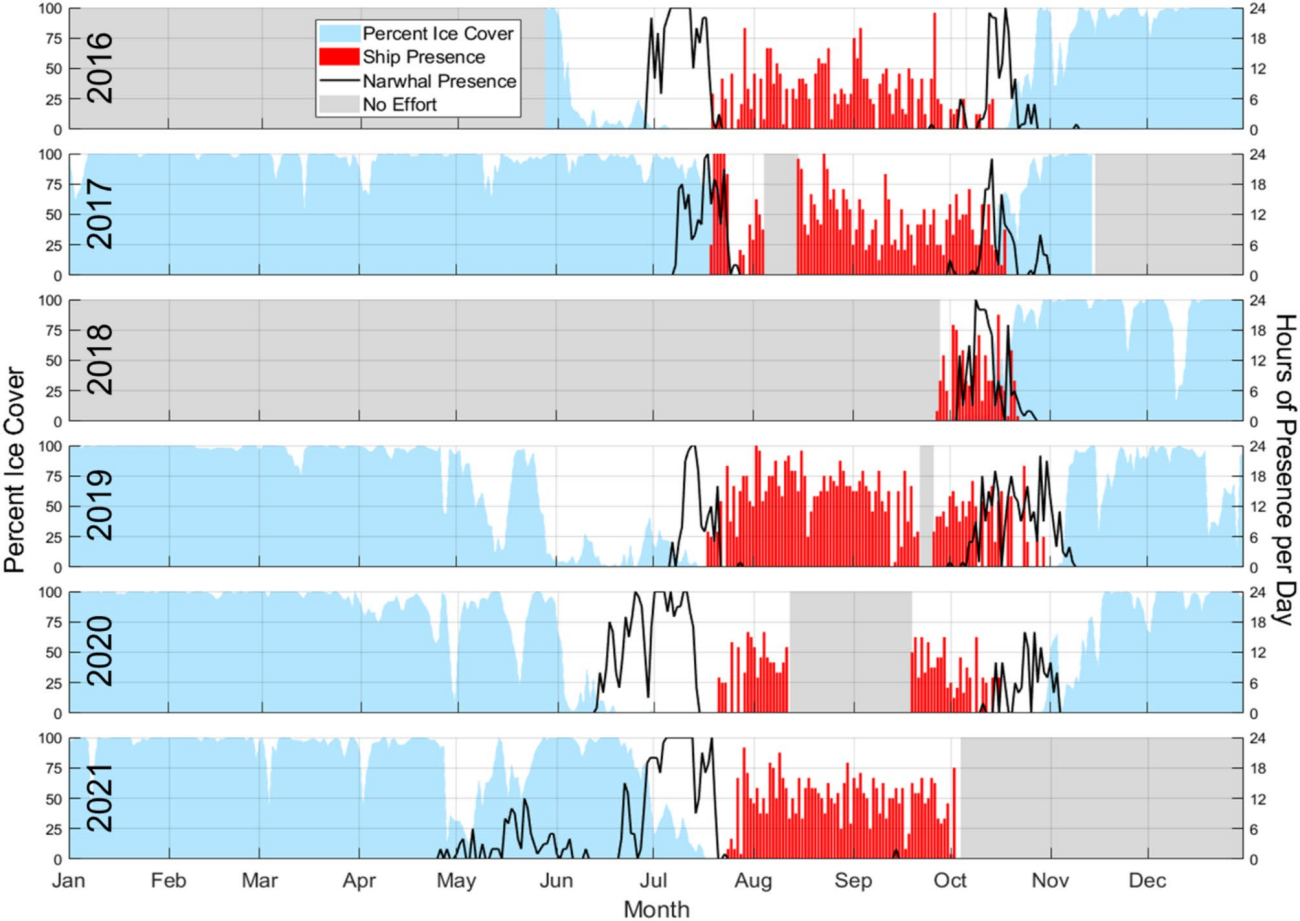
Time series of narwhal detections, sea ice cover, and ship presence for all recording periods. Black lines denote narwhal acoustic presence, and the red bars are ship presence within a 40 km radius of the recording location, both in hours present per day. Blue filled area is the mean daily percent ice cover within 10 km of the recording site. Gray areas indicate periods with no acoustic recording effort.

Narwhal echolocation clicks were detected at site EE from late April to mid November between 2016 and 2021 (Fig. 2). Detections peaked in July and October, coinciding with the annual sea ice breakup and formation periods. In contrast, narwhal echolocation detections were sparse or absent when the mean daily sea ice concentration (SIC) within a 10 km radius of the recording site exceeded 80% (typically from November to June), or during ice-free months from early August to late September.

To investigate annual patterns of narwhal presence at site EE, Generalized Estimating Equations (GEE’s) were applied to hourly time intervals. Two separate models were constructed to better understand these patterns: the first model examined generalized temporal variables, such as day of year and year, while the second focused on specific environmental variables, including sea ice concentration. The two-model approach was adopted to reveal the influence of temporal and environmental factors on narwhal presence without introducing multicollinearity into a single model. Seasonal and environmental variables may be strongly related in an Arctic environment.

**Fig. 3:**
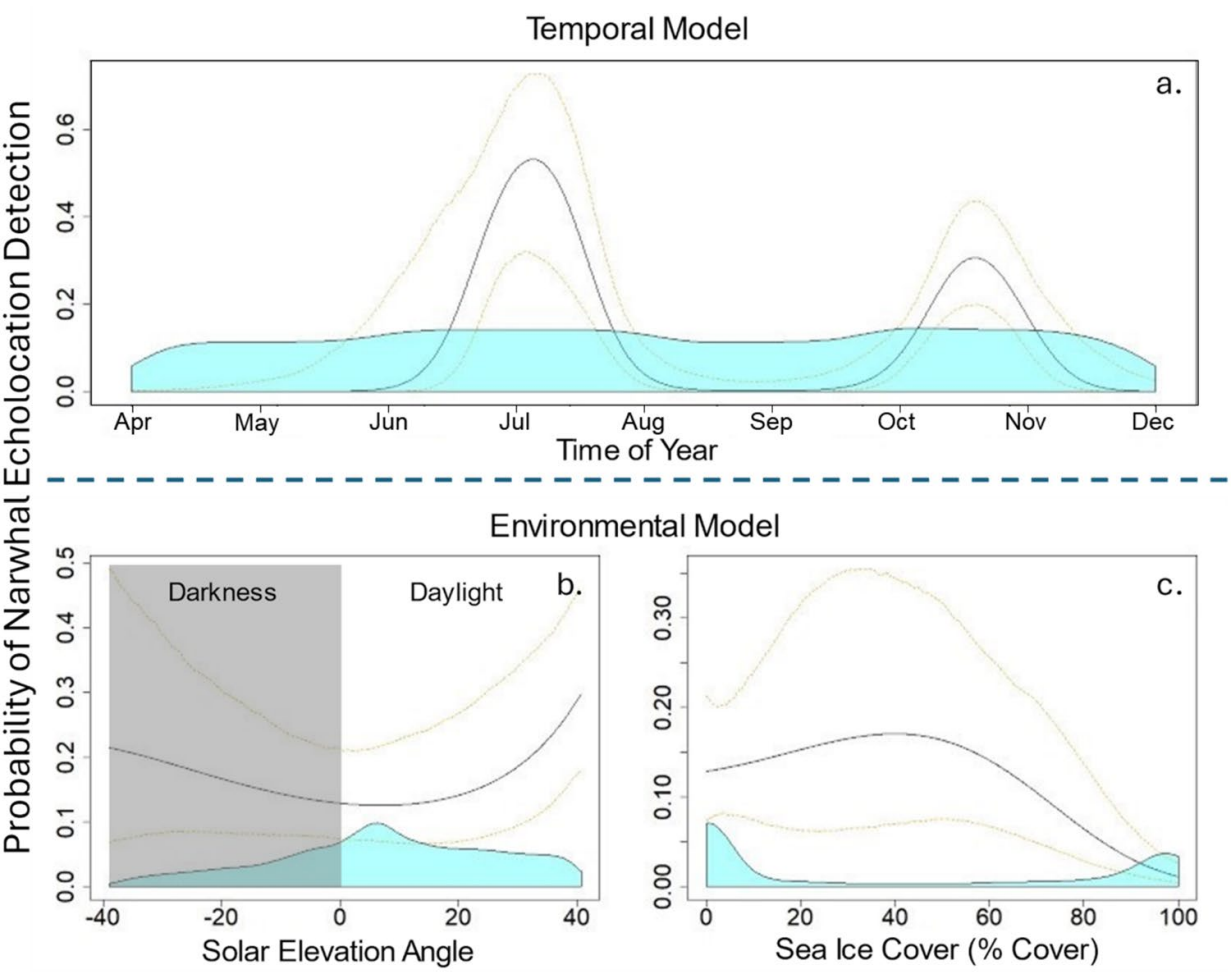
Key predictive relationships from two GEE models based on temporal and based on environmental variables respectively. Plots show hourly probability of narwhal acoustic presence based on (**a**) time of year (temporal model), (**b**) solar elevation angle relative to the horizon, and c) percent ice cover (environmental model). For the environmental model (**b** & **c**), predictions represent the relationship between each predictor variable and narwhal presence while other predictor variables remain fixed at specific values: 0% ice cover, and a solar elevation angle of 0 degrees (median). The black line indicates the model estimate, and yellow dotted lines are 95% confidence intervals. The blue shading at the base of the plot represents the relative distribution of the predictor variable values used in the model.

The mean duration of continuous narwhal acoustic presence during individual detection events, defined as periods of presence not separated by more than 15 min, was 108.6 min (SD = 369.4 min, median = 29 min, max = 5.90 days) across all continuous recording periods (Table 1). In the annual temporal model, day of year was significant in predicting the presence of narwhals at the recording location (Wald *χ*^2^ = 19.8, *p* < 0.001; Fig. 3a). Variability between years, however, was not statistically significant, highlighting a strong seasonal signal without substantial interannual fluctuations. Narwhals were consistently detected from June to late July, a period coinciding with the regional season of late spring (May 15 to July 15; *Upingaaq*^19^). Narwhal echolocation was detected on a greater proportion of days in early fall than during late spring, with presence on 64.5% of days in October compared to 55.5% of days in July on average (Fig. 2). In the annual environmental model, sea ice concentration (SIC) was significant in predicting narwhal acoustic presence (Wald *χ*^2^ = 81.3, *p* < 0.0001), with a higher probability of detection when the ice cover was below 85%, followed by a decline at greater SIC values (Fig. 3c). In May 2021, sea ice concentrations near the recording location were lower and detections began earlier than in other years. The proximity of the recording location to the floe edge—the boundary where stationary sea ice attached to land meets drifting pack ice —was also closer during 2021 than in other years.

**Table 1:** Acoustic recording periods used in this study from 2016 to 2021.

Deployment	Effort start time	Effort stop time	Recording duration (days)	Latitude	Longitude	Depth (Meters)
1	05/28/2016 20:25:00	10/05/2016 15:35:17	130	72.724 N	76.233 W	657
2	10/05/2016 22:00:00	08/04/2017 09:28:34	302	72.724 N	76.231 W	670
3	08/15/2017 00:00:00	01/30/2018 05:09:50	168	72.725 N	76.230 W	670
4	09/27/2018 22:00:00	09/21/2019 15:59:12	359	72.729 N	76.225 W	670
5	09/26/2019 00:00:00	08/12/2020 00:00:00	321	72.729 N	76.224 W	640
6	09/19/2020 00:00:00	08/13/2021 00:00:00	328	72.729 N	76.220 W	638

Periods were included if acoustic recordings, sea ice concentration estimates, and ship AIS data were obtained. Deployment three operated on a 75% recording schedule, capturing 15 min of data, followed by a 5-min pause in recording. All other deployments were recorded continuously.

In the annual environmental model, a seasonal diel pattern emerged in narwhal acoustic presence. The angle of the sun relative to the nautical horizon (*i.e.* solar angle) had a significant relationship with the probability of detecting narwhal echolocation (Wald *χ*^2^ = 140, *p* < 0.00001). Predicted probabilities of narwhal presence are highest when the sun is either well above or well below the horizon, with the lowest probabilities occurring when the sun is near the horizon (Fig. 3b), possibly due to narwhal presence coinciding with seasonal periods of differing patterns in solar elevation. During late spring and early summer in Eclipse Sound, daylight is continuous, with the sun remaining above the horizon. As fall progresses and the sun dips below the horizon for extended periods.

### Annual ship presence

**Fig. 4:**
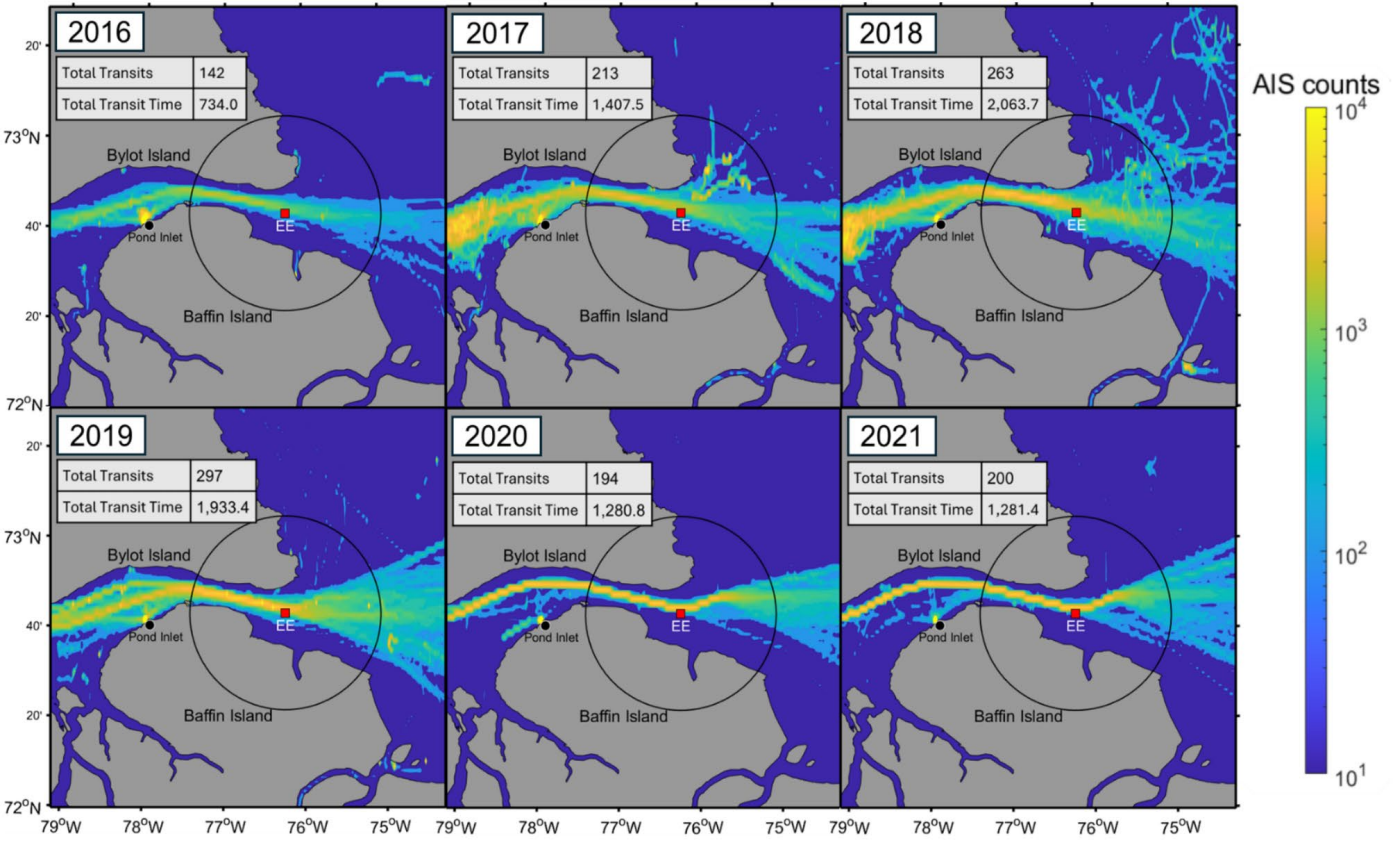
Ship AIS location density from 2016 to 2021. The heat map shows the log-transformed AIS counts per km^2^. The black circle around site EE (red square) indicates the 40 km analysis radius. Ship location density increases substantially from 2016 to 2019. The number of ship transits and total ship presence, measured in hours, were determined using AIS data for all days of the year.

The annual number of ship transits past the recording location and the total time with least one ship within a 40 km radius of the site generally increased from 2016 to 2019, then remained relatively constant in 2020–2021 (Fig. 4). The shipping season, defined as the period of time between the first and last detected ship of the year, occurred predominantly during ice-free periods. Ship transit numbers and total transit time were lowest in 2016, which also had the shortest shipping season at 88 days. Between 2016 and 2019, the annual number of transits passing within 15 km of the recording site more than doubled, increasing from 142 to 297. The longest shipping season occurred in 2019, spanning 105 days total. In 2020 and 2021, shipping traffic decreased to levels similar to 2017, likely due to the COVID-19 pandemic, which disrupted global shipping and reduced passenger ship and pleasure craft traffic in the Canadian Arctic^20^.

### Behavioral responses of narwhal to ship proximity

**Fig. 5:**
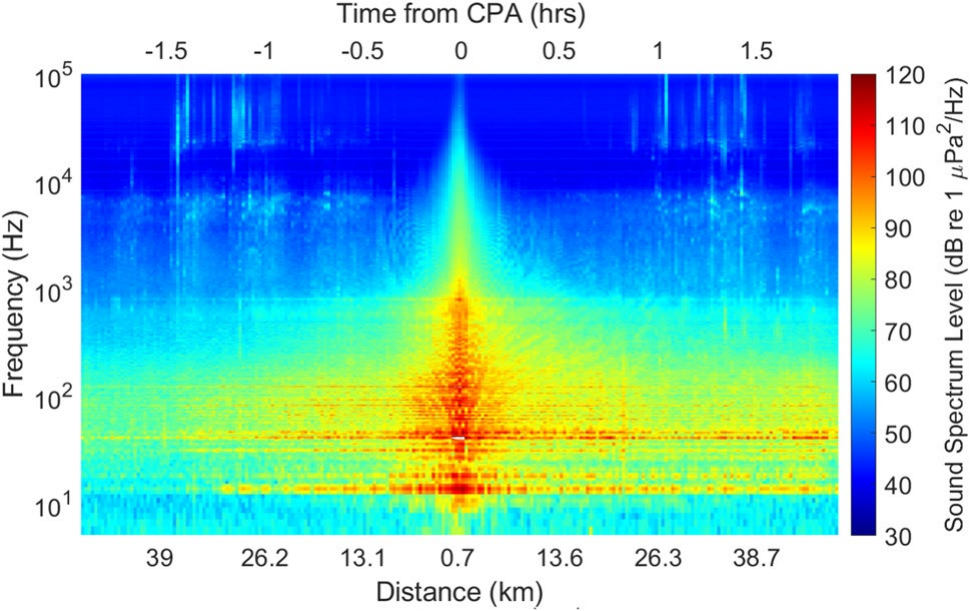
Ship transit event with acoustic presence of narwhals. Canadian Coast Guard Icebreaker, *Terry Fox* on Oct 4, 2018, traveling at speed over ground 14 knots past the recording location at the eastern entrance to Eclipse Sound. Long-term spectral average (LTSA) plot showing ship distance from the recording location, time with respect to closest point of approach (CPA), and received sound pressure spectrum levels (color bar) for frequencies from 20–20,000 Hz during a 4-h ship transit window.

A representative time period with narwhal acoustic presence during a ship passage is shown in Fig. 5, illustrating the acoustic occurrence of narwhal echolocation clicks during the period when a ship (Canadian Coast Guard Icebreaker Terry Fox) was transiting past the recording location. The long-term spectral average (LTSA) of a six-hour time window including a ship passage near the recording site shows a typical pattern: narwhal echolocation clicks are present as the ship approaches, then diminish around the closest point of approach, and resume as the ship travels away from the recorder. Across all recordings from 2016–2021 in which narwhals were acoustically detected while a ship was within 40 km of the recording location, detections ceased in 15.4% of cases when the ship was within 30 km, 30.8% within 20 km, 42.3% within 15 km, 46.2% within 10 km, and 76.9% of cases when the ship came within 5 km.

#### Model results: July

**Fig. 6:**
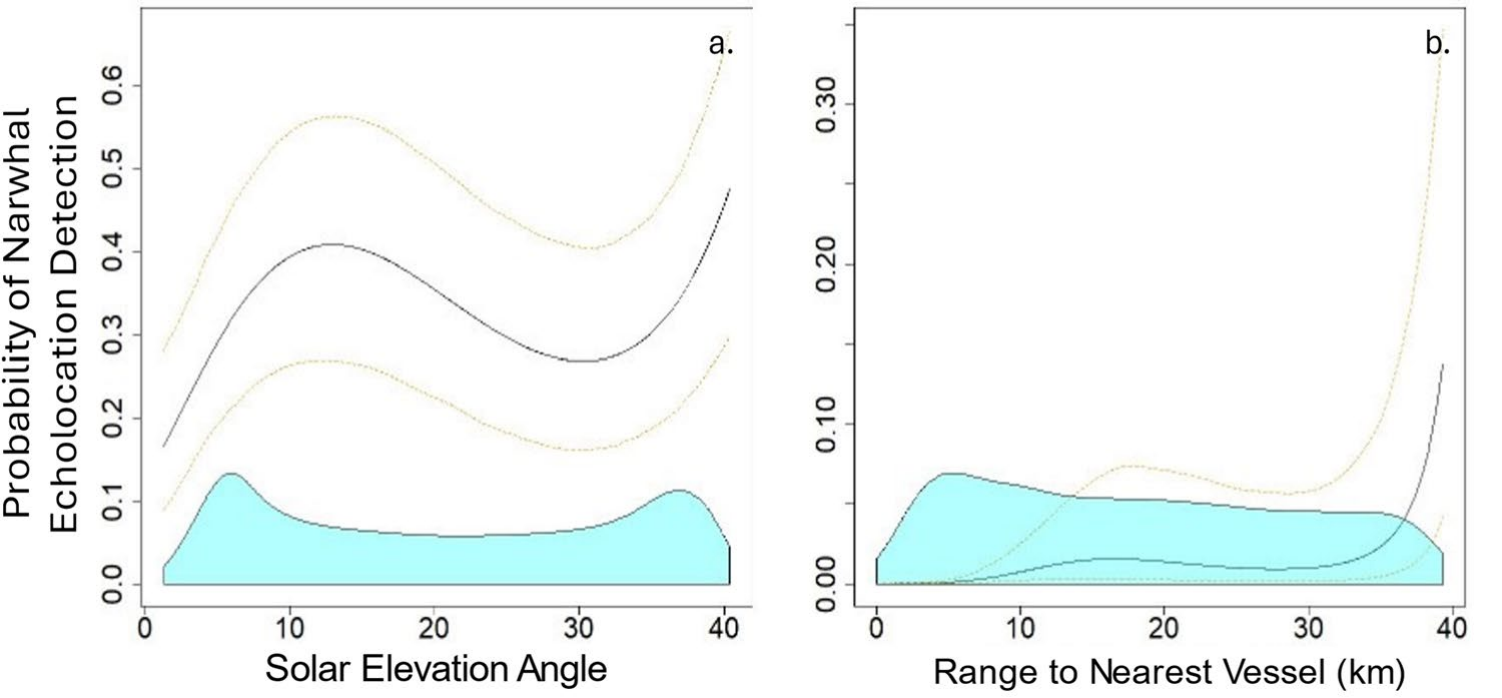
GEE model results showing the probability of narwhal acoustic presence during July (in five-minute time bins) relative to the predictor variables: (**a**) solar elevation angle and (**b**) range to nearest ship. The reference state set for solar elevation angle included no ship presence, and the median solar elevation for the range to the nearest vessel. The black line indicates the model estimate, and yellow dotted lines are 95% confidence intervals. The blue shading at the base of the plot represents the relative distribution of the predictor variable values used in the model.

No significant relationship was found between narwhal acoustic presence and sea ice concentration in the month of July (Fig. 6). This lack of correlation is likely due to the near-constant presence of narwhals at the floe edge, typically within 10 km of the recording location during the annual period of sea ice retreat and break-up. The mean daily sea ice concentration estimates for July includes both landfast and pack ice, which may confound discovery of related patterns in narwhal acoustic presence. This analysis does not account for the distinct influences of these two ice types as separate factors. In July, narwhal acoustic presence varied significantly with solar elevation at the site (Wald *χ*^2^ = 22.56, *p* < 0.0001). A peak predicted detection probability occurred when the sun was between 10 and 20 degrees above the nautical horizon, although solar angle was mostly between 5 and 40 degrees above the horizon during July (Fig. 6a).

During all recording periods from 2016 to 2021, only 6.5% of acoustic recording days in July (n = 11/155 days) showed overlapping presence of ships and narwhal echolocation. Regional ship traffic is minimal in July due to landfast and pack ice that restricts navigation in Eclipse Sound and Baffin Bay. During all July recording periods, 11 of 139 ship transit windows (i.e. ship within 40 km of the recorder) coincided with narwhal acoustic presence. The number of daily ship transits in July analyzed ranged from zero to eight. Despite minimal instances of exposure to ships in July, the probability of narwhal acoustic presence significantly decreased as ships approached closer to the recording site (Fig. 6b; Wald *χ*^2^ = 2587.48, *p* < 0.0001).

#### Model results: October

**Fig. 7:**
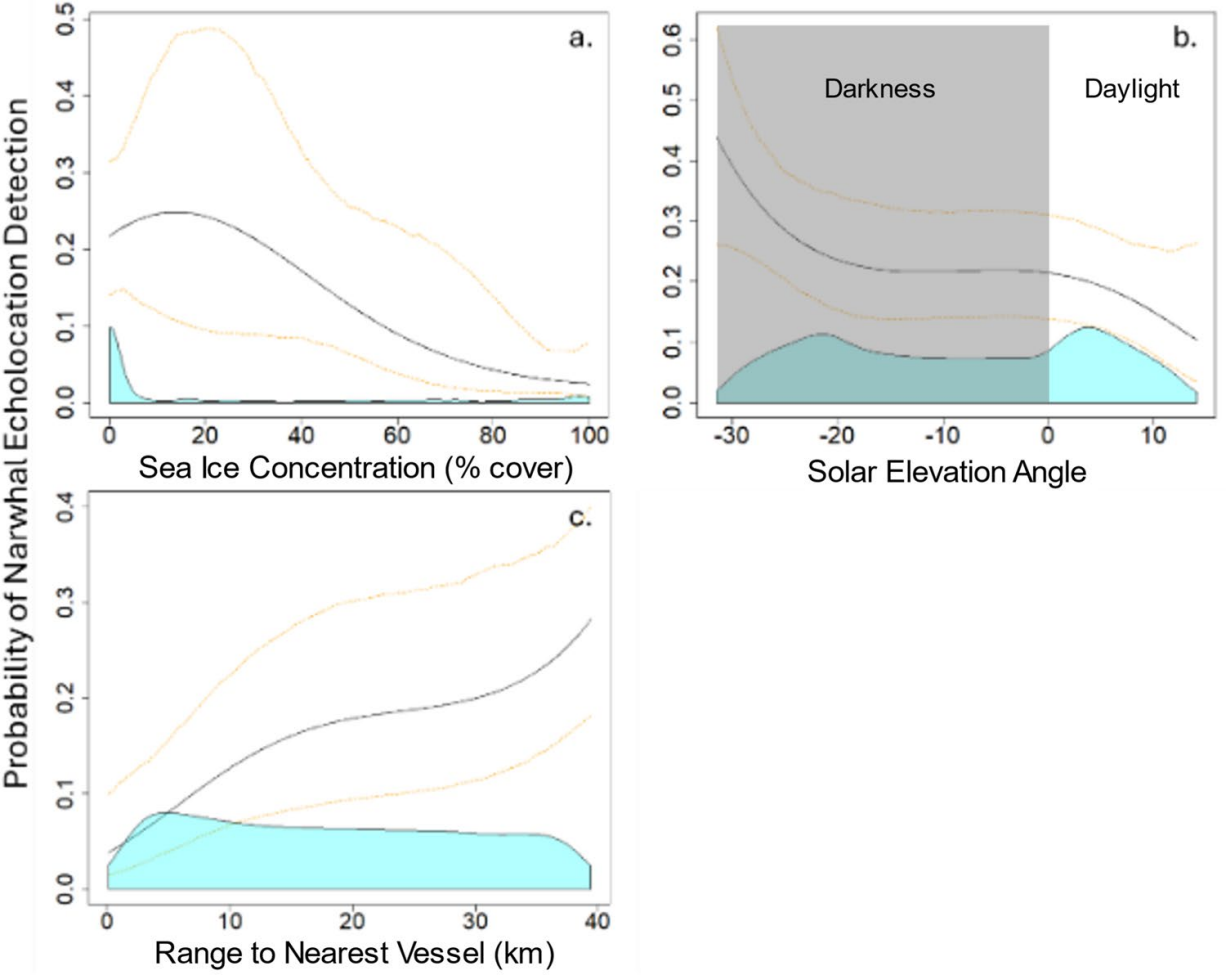
GEE model results showing the probability of narwhal acoustic presence during October (in five-minute time bins) relative to predictor variables: (**a**) percent ice cover, (**b**) solar elevation angle relative to the horizon, and (**c**) distance to the nearest ship. There is a higher probability of narwhal presence at night, at mid to low sea ice concentration levels and at greater distances from ships. All plots shown are set to specific reference conditions of 0% ice cover, the median solar elevation angle, and no ship presence. The black line indicates the model estimate, and yellow dotted lines are 95% confidence intervals. The blue shading at the base of the plot represents the relative distribution of the predictor variable values used in the model.

Sea ice concentration (SIC) was a significant predictor of narwhal acoustic presence in October, with a decline in the probability of detection when SIC exceeded 80% (Wald *χ*^2^ = 19.0, *p* < 0.001; Fig. 7a). Additionally, significant diel patterns were observed, with higher detections of narwhal echolocation (Wald *χ*^2^ = 19.1, *p* < 0.001) during nighttime periods when the solar elevation angle was below the horizon (Fig. 7b).

The greatest overlap in narwhal and ship presence occurred during October, coinciding with the early period of annual ice advance in the Eclipse Sound region (Fig. 2). Of all 2016–2020 October days with acoustic recordings, 27% (n = 41/155) had overlapping presence of ships and narwhal echolocation. A total of 59 of 179 October ship transits coincided with narwhal acoustic presence while ships were within a 40 km radius of the recorder, with daily number of ship transits ranging from zero to six. Narwhal acoustic presence was significantly related to the range to the nearest ship (Wald *χ*^2^ = 50.4, *p* < 0.00001) (Fig. 7). The probability of narwhal acoustic detection decreased as distance to the nearest ship decreased, with a notable change in slope around 20 km distance between the ship and recording location (Fig. 7c, tables S4-5). The 95% confidence intervals also exhibit this pattern at about distances of 20 km (upper bound) and 12 km (lower bound).

## Discussion

The probability of narwhal acoustic presence at the eastern entrance to Eclipse Sound is strongly influenced by time of year, environmental conditions, and ship proximity. Seasonal peaks in acoustic presence align with the periods of sea ice breakup in July and formation in October, reflecting distinct migratory patterns that are consistent with Inuit knowledge of narwhal movements in the region. In the month or more prior to breakup, narwhals are associated with the floe edge, typically within 10 km of the recording site. After breakup, they move into the interior inlets and bays of Eclipse Sound, remaining until ice formation begins in early fall. While narwhal echolocation detection in October is significantly associated with mean daily sea ice concentration, the reliance on daily averages limits the ability to resolve finer-scale temporal patterns in their presence in relation to sea ice.

This study is the first to document season-specific relationships between solar angle and narwhal acoustic presence, finding higher acoustic activity during hours of low sun angle in July and when the sun is below the horizon in October. This relationship may reflect differences in habitat usage between seasons, diel foraging patterns, or strategies to avoid visual predators. Inuit hunters engaged in subsistence harvest of narwhal within 40 km of the recording site report that more narwhals are caught near shore during nighttime than daylight in early fall, a pattern attributed to increased narwhal presence near shore at night during this period. Understanding these diel patterns will be valuable for future research aiming to quantify the acoustic presence of narwhals and other Arctic marine mammals.

During seasonal transition periods, narwhals in Eclipse Sound are exposed to large ships transiting through their habitat. The probability of detecting narwhal echolocation clicks decreases as ship proximity increases, with the most pronounced effect in October at distances less than 20 km from the recorder. Narwhals appear to either move away or stop vocalizing as ships approach, with acoustic detections resuming at similar distances after ships have passed (Fig. 5). A similar, but less marked, pattern was observed in July, likely reflecting fewer ship transits during that month, although narwhal are present at the study location.

Ship proximity was selected as the predictor variable for these analyses to facilitate comparisons with previous studies of narwhal responses to ships^9,17^. However, narwhal behavioral responses are also likely influenced by sound exposure levels. Broadband sound pressure level (SPL_bb_; 20 to > 10 kHz), is a standard metric for assessing behavioral impacts of underwater noise on marine mammals, with behavioral responses in narwhal and most odontocetes generally predicted at received SPL_bb_ of 120 dB re: 1 μPa or higher^21,22^. Prior analysis of 2016–2019 ship noise at the study site showed SPL_bb_ for most ships was well below this threshold at distances of > 5 km and ranged between 94 and 109 dB re 1 μPa at a distance of 20 km^16^. Our findings indicate that narwhal responses occur at ship distances well beyond 5 km, suggesting the generalized 120 dB re: 1 μPa threshold may underestimate their behavioral sensitivity to noise at the eastern entrance to Eclipse Sound.

Underwater noise from ships at distances > 10 km from the recording location is concentrated in the 20–200 Hz frequency range^16^ (e.g. Figure 5), below frequencies narwhals are presumed to perceive based on generalized audiograms for high-frequency cetaceans (HFC)^22^. However, Inuit observations consistently report that narwhals have highly sensitive hearing, enabling them to detect ships from great distances^4–7^. The significant decrease in narwhal acoustic detections when ships are within 20 km of the recorder during October suggests that generalized assumptions about their hearing^21,22^ may underestimate both their auditory sensitivity below 1 kHz and the broadband sound pressure level relevant for assessing noise impacts. These findings caution against relying solely on generalized auditory weighting functions when evaluating noise impacts on narwhals.

For instance, a previous study conducted in the Eclipse Sound region during 2018–2019 applied HFC auditory weighting to received sound pressure spectrum levels during ship transits. Based on these analyses, the authors suggested that narwhals were "unlikely to clearly perceive shipping noise unless ships were in close proximity (< 3 km) and ambient noise levels were low”^23^, and predicted no biologically significant difference in broadband sound pressure levels with or without ships present within 15 km of the recorder. These predictions of narwhal auditory sensitivity are not consistent with published received sound pressure spectrum levels at the behavioral response distances observed in our study.

While our results highlight the influence of environmental factors and ship presence on narwhal acoustic presence and behavior, it is important to consider other factors that may play a role in their presence or detectability. For example, the presence^24^ and predation activity of killer whales in the Eclipse Sound region, may influence narwhal movements on seasonal and shorter timescales. Small boat traffic and subsistence hunting of narwhal do not generally occur at this study site during October. However, these could be important predictors of narwhal presence and behavior in other areas and at this study location during late-spring when subsistence harvest occurs at the floe edge. Exploring connections between narwhal presence and broader regional habitat factors may clarify how conditions beyond the immediate study area influence narwhal movements and distribution patterns not solely explained by local factors such as sea ice.

Sea ice dynamics and physical characteristics at finer spatial and temporal scales than examined here may affect narwhal acoustic detection. Large ice floes, which frequently pass over the recording site during breakup, may temporarily reduce the proximity of echolocating narwhals. While narwhals readily navigate smaller floes^1,14^, this study does not account for the effects of individual floe size, ice type, or variations in sound transmission loss due to ice coverage. Additionally, differences in detection probability related to behavioral state (e.g., foraging vs. transiting) were not addressed.

Interpreting periods without narwhal acoustic detections requires consideration of several factors. Reduced acoustic detections with increasing ship proximity may reflect narwhal behavioral responses, but could also be caused by signal masking resulting from environmental or ship noise. Masking was unlikely to affect narwhal acoustic detection probability in this study. Echolocation click detection was limited to frequencies above 15 kHz; and prior analyses show ship noise at these frequencies is rarely measurable beyond 3 km^16^. The likelihood of ship noise interference with acoustic detection is very low at the estimated 5.3 km maximum detection range and 120 dB_pp_ minimum click received level thresholds applied.

Although the present study focuses on narwhal proximity to ships, assessing the underwater noise levels to which these animals are exposed during ship transits is also important. Further analyses of narwhal responses to underwater radiated noise from different ship types and individual vessels could provide more detailed information for resource management and decision-making. In the absence of direct underwater hearing measurements for narwhals, behavioral observations in response to varying levels and frequencies of sound exposure offer an opportunity to improve our understanding of narwhal hearing and perception.

A limitation of the current approach is reliance on the distance between the ship and hydrophone, rather than the actual distance between the ship and narwhals. The 120 dB peak-to-peak received level threshold for narwhal echolocation clicks enables a simplifying assumption in this study, that narwhal acoustic presence is counted only when animals are within a 5.3 km radius of the hydrophone. Refining received sound level measurements, categorizing ships by acoustic signature, and developing more accurate methods for estimating narwhal positions relative to noise sources would advance our understanding of their responses to environmental factors and underwater noise from ships.

This study provides new insights into narwhal behavioral responses to environmental variability and ship traffic in Eclipse Sound. These findings underscore the importance of developing species- and region-specific understanding of narwhal relationships with environmental factors, such as sea ice, and behavioral responses to ship traffic. Such detailed knowledge will help inform management and decision-making related to shipping within the broader context of environmental variability and change in the Arctic.

## Methods

### Acoustic recordings

Underwater acoustic recordings were collected year-round in the Eclipse Sound region, at a location 60 km east of the community of Pond Inlet, Nunavut, Canada. The recording system was located at the eastern entrance to Eclipse Sound (EE, *Sanniruup Saangat*; Fig. 1), positioned directly under the most common track line for large ship traffic entering and exiting the region. Acoustic data were collected during all months of the year at site EE using High-frequency Acoustic Recording Packages (HARPs)^25^, recording continuously at a sampling rate of 200 kHz. Audio data was recorded from May 2016 to October 2021 (Table 1).

The HARPs were anchored to the seafloor, with the hydrophone sensor suspended approximately 20 m above the bottom. The hydrophone consisted of two stages, one for low-frequency (< 2.5 kHz) and one for high-frequency (> 2.5 kHz). The low-frequency stage was composed of six cylindrical transducers (Benthos AQ-1) providing a hydrophone sensitivity of -187 decibels (dB) re: V/μPa and with an additional 55 dB of preamplifier gain. The high-frequency stage consisted of a spherical omni-directional transducer (ITC- 1042; www.itctransducers.com) with an approximately flat frequency response of − 200 dB root mean squared (RMS) re 1 V/μPa between 1 and 100 kHz with an additional 50 dB of preamplifier gain. The hydrophones used from September 2018 to September 2021 had the same frequency range but employed a single spherical transducer (ITC-1042). All hydrophones were calibrated at the Scripps Institution of Oceanography, enabling conversions to absolute sound pressure levels. The acoustic calibration of each hydrophone facilitated direct comparison of received sound levels and acoustic detections between deployment years and instrument configurations.

### Identification of narwhal echolocation clicks

Narwhal echolocation clicks were identified in acoustic recordings through a four-step analytical process for signal detection and classification: (1) automated impulse detection, (2) removal of spurious detections based on spectral and temporal characteristics, (3) unsupervised clustering to identify acoustically similar signals, and (4) manual review and labeling of detections by a trained analyst. All analysis was carried out using custom software^26,27^ written for MATLAB (mathworks.com).

During the initial phase of data analysis, signals consistent with narwhal echolocation clicks were detected from 2016–2021 recordings at site EE. An energy-based detector was utilized to locate impulsive signals fitting specific criteria^28,29^. The spectrum of each detected impulse was computed using a 200-sample window paired with a Hanning window. This produced a sound pressure spectrum level measurement, with 500 Hz resolution. The band-pass filtered pressure time series of every detected impulse was archived, to facilitate subsequent in-depth analyses. A high-pass filter of 5 kHz was applied to reduce false detections from impulsive sounds below the frequency range for narwhal. Signals with a peak-to-peak received level below 118 dB re: 1 μPa were discarded. Additional constraints were applied to the remaining signals: impulse durations between 30 and 1200 microseconds; peak frequencies ranging from 5 to 100 kHz; and energy envelope ratios bounded by -0.5 (minimum) to 0.9 (maximum). Additionally, a digital clipping threshold was established to exclude signals with amplitudes greater than 98% of the maximum representable 16-bit value.

In the second step, a threshold method was applied to remove spurious detections. Echolocation inter-click intervals (ICIs) spanning values greater than 0.5 s and below 2 ms were removed from the dataset. The focus was narrowed exclusively to click trains, which were characterized as sequences encompassing at least 10 consecutive echolocation clicks that fit within the prescribed ICI range.

In the third step, detections were grouped by a two-stage clustering algorithm. Using an unsupervised learning approach rooted in impulse spectral shape and ICI distributions, detected signals were categorized into distinct impulse types^26^. For this phase, only impulses with peak frequencies between 15 and 90 kHz, received levels exceeding 118 dB peak-to-peak (dB_pp_) were conserved for the tertiary analysis stage. The algorithm was tuned with a pruning threshold of 90% and adopted a time bin size of 5 s, mandating a minimum cluster size of 50. This strategy was designed to filter out erroneous detections from the initial analytical step. Unique impulse types were discerned by 10 iterations through the detection time series windows for each 5-min time interval. Detections were collated within each time bin, based on spectral and ICI similarities.

The fourth stage of analysis involved manual review and final labeling of the detection set to remove false positive and add false negative detections of narwhal clicks. In this final stage, an expert analyst (JPE) used *detEdit*^26^, a MATLAB open-source software, to discard false detections caused by transiting ships, wind, rain, sea ice, and echolocation clicks of sperm whales, occasionally present during October. One-minute segments of data were examined using comparative panels showing long-term spectral average, received level and time between detections, as well as spectral and waveform plots. Spectral and waveform displays of selected detections were compared with established characteristics of narwhal echolocation^28^, ensuring the accuracy of the click identification. Additionally, peak frequency as a function of received level was considered, as narwhal echolocation click frequency spectra vary with received level^28^. Only narwhal click detections with peak-to-peak (pp) received level above 120 dB_pp_ were retained for further analysis.

A conservative 120 dB_pp_ threshold was estimated to set a maximum detection radius of approximately 5.3 km^30^ assuming a narwhal echolocation click source level of 215 dB_pp_^31^_._ Sound propagation was evaluated at 20 kHz using an absorption coefficient of 3.82 dB_pp_ per kilometer^32^, average deployment depth of 660 m, 35 PSU salinity, PH of 8 and temperature of 1 degree Celsius. We assumed that seasonal changes in surface temperature and salinity were unlikely to strongly influence detection range for narwhal echolocation, which is typically produced below the stratified surface layer at depths greater than 500 meters^33^.

### Sea ice measurements

Average daily sea ice concentration (SIC) and sea ice thickness were estimated to evaluate the influence of sea ice on the presence of narwhal echolocation. Advanced Microwave Scanning Radiometer 2 (AMSR2) data with a 3.125 km spatial resolution were obtained from the University of Bremen^34,35^. Utilizing the Windows Image Manager (WIM) and Windows Automation Module (WAM) software tools^36^, median daily sea ice concentrations were estimated for all AMSR2 grid values within a spatial mask of a 20 km radius centered on our recording location. To reduce the effects of terrestrial snow and ice on sea ice estimation, an additional mask was applied to remove all pixels within one km distance from the nearest shoreline. The daily arithmetic mean, variance, and median of sea ice concentrations, expressed as a percentage of the total mask area, were computed using WAM. The median value for the sea ice measurements was chosen for further analysis because the passive microwave data had greater ice concentration values at each grid point than expected, potentially due to the proximity of the recording site to nearby land on Baffin and Bylot Islands. This issue was particularly noticeable during ice-free periods in the late summer, consistent with the product’s estimated increase in absolute error as SIC approaches 0%^37^.

Daily thin Sea Ice Thickness (SIT) measurements from L-band sensors aboard Soil Moisture and Ocean Salinity (SMOS) & Soil Moisture Active Passive (SMAP)^38^ missions were obtained from the University of Bremen at a spatial resolution of 12.5 km. Thin SIT values can only be reliably collected during sea ice formation periods, so data were only obtained for the month of October over all on effort recording periods (Table 1). Daily gridded values were obtained for a spatial region with a 20 km radius centered on our recording location both to match the resolution of our SIC values and to obtain data over the proper resolution of both satellites (40 km)^38^. Analysis of SIT data was conducted using MATLAB and daily median values were selected among the pixel values within our 20 km of our recording site.

### Ship AIS data analysis

AIS ship location and operational data were used to investigate the relationship between narwhal acoustic presence and proximity of ships to the recording location. Satellite AIS data were obtained from Spire (spire.com/maritime) and included time, latitude, longitude, speed over ground (SOG), heading course over ground (COG), operating draft, Maritime Mobile Service Identity (MMSI) number, ship name, ship type, and cargo class. Information was also obtained on ship gross and deadweight tonnage from Lloyd’s Registry of Ships (lr.org/en/lrofships). Ship transit windows were defined as periods during which a ship was continuously present within a 40 km radius of the recording location, with the Closest Point of Approach (CPA) occurring within a 15 km range of the acoustic recorder. Minimum Range of the Nearest Vessel (RNV) to the location of the recording site was computed for each minute. To facilitate comparisons with previous studies, a 40 km radius around the recording location was selected for analysis of narwhal behavioral responses to ship traffic. A prior study from eastern Greenland identified that the impact of ship noise on narwhal behavior was detected within a maximum distance of 40 km from the ship^9^. Additionally, this radius is consistent with a previously published ship noise analysis in the same acoustic recordings collected at site EE^16^. For the purpose of this study, "continuous ship presence" was defined as periods of presence with a maximum gap of 60 min between successive AIS position updates during each ship transit window. Ship speeds of up to 18 nautical miles per hour (knots) were observed during these transits. We defined ‘loitering ships’ as those moving at velocities under 4 knots. Transit windows including loitering ships were not included in this analysis. We interpolated all ship locations to a temporal resolution of 5 s to fill any gaps in AIS data due to periods with no satellites within range. All the interpolated AIS data were aggregated and averaged to a 5-min temporal resolution for statistical analysis.

### Statistical modelling

Our statistical analysis was divided into two main parts: (1) narwhal seasonal acoustic presence during May 1 through November 30, and (2) their acoustic presence in relation to ships during two one-month periods of co-occurrence of ships and narwhal clicks. We analyzed seasonal narwhal acoustic presence (binary, with 1 as present and 0 as absent) at an hourly temporal resolution, excluding the period from December 1 to March 31 due to the absence of narwhal acoustic presence during times of complete ice coverage. Two models were used for these annual periods: one examining generic temporal variables to describe narwhal presence at different time scales and the other examining environmental variables to investigate what conditions drive variability of presence. This was done to give better context for the year-round presence without including collinear predictors (sea ice & day of year) in the same model. A flow diagram of the statistical modelling approach can be found in figure S1. For seasonal analysis including environmental variables, the percent ice cover, sun elevation angle, day of year, and year were considered for explaining narwhal acoustic presence. Solar elevation angle (accounting for atmospheric refraction) was obtained for the coordinates of our recording location based on the formulas used in the NOAA Solar Calculator (gml.noaa.gov/grad/solcalc)^39^. Solar angle was selected as a predictor variable over normalized time of day due to extended periods of constant daylight or darkness at this latitude.

July and October were identified as the months with the highest annual overlap between narwhal acoustic presence and ship activity within 40 km of the site (Fig. 2). To investigate narwhal responses to noise exposure from individual ship transits, we analyzed narwhal acoustic presence, range of the nearest non-loitering ship from the recording site, and other potential predictor variables at a five-minute time resolution during these months. Environmental variables considered for inclusion as predictors of narwhal presence were percent ice cover and solar elevation angle. Thin sea ice thickness was also included in October only, as this parameter is only measured during freeze-up periods due to physical and radiometric limitations of the satellite sensors^38^. All data processing and preliminary analyses were performed using MATLAB, while the statistical analyses were conducted in R.

Data exploration of all predictor variables provided information on both the potential distributions and relationships of each variable to narwhal echolocation presence (Figure S2). Pearson correlation coefficients were computed to assess correlation between explanatory variables, with thresholds above 0.6 or below -0.6 indicating strong correlation^40,41^ (Figure S3). Generalized Linear Models (GLM) were fitted with all predictors to test for multicollinearity using a generalized variance inflation factor (GVIF) analysis with the function *vif* from the R package *car*^42^. Starting with a full model with all variables fitted in a GLM, variables with high collinearity with a cut-off value of 3.0^43^ were removed one by one using a stepwise procedure. This resulted in the removal of year from the annual analysis, year in the July analysis, and sea ice thickness in the October analysis (Table S1). While we acknowledge that Lasso or Ridge regression could also address multicollinearity in a global model, these methods are not well-suited for the autocorrelated nature of our time-series data, which is critical given the temporal structure of narwhal responses to ships.

This study uses a multi-year time series of acoustic recordings and candidate predictor variables to examine the fine-scale effects of ships and environmental variables on narwhal acoustic presence, which can last from minutes to hours. To model these effects, we employed Generalized Estimating Eqs. ^44^ (GEEs), which allowed for estimates of population-average parameters representing effects from correlated data with asymptotically correct standard errors^45–49^. The correlation of narwhal acoustic presence to itself was estimated using the autocorrelation function, *acf* function from the *stats*^50^ package in R, and it ranged between 2,522 (July) to 1,797 (October) 5-min bins (Figure S4). Binomial GEE models were fit using the function *glmgee* from the R package *geepack*^51^ with a logit-link function, and best correlation structure, independent or autoregressive was chosen based on the lowest value under the Quasi-likelihood under the Independence model Criterion^52,53^ (QIC). Predictor variable *Year* was treated as a factor variable in the model, and the periodic variable *Day of year *was treated as cyclic splines limited to 4 degrees of freedom using the *mspline* function in the *splines2* package^53^ to help interpretability of the seasons and daylight phases. The remaining non-collinear variables were evaluated individually as a linear or smooth term for inclusion in models using the lowest Quasi-likelihood Information Criterion (QIC) value^52,53^ from the R package *geepack*^51^ using the determined cluster size of narwhal presence autocorrelation from the previous step. Smooth terms were built as a cubic B-spline to allow for greater flexibility in the interaction with the response. The *bs* function from the *splines*^54^ package was used with the default settings to fit a third-degree polynomial with no inner knots. To account for the fact that ship proximity is only available when a vessel is present, ship presence/absence was included as an interaction term with ship proximity to accommodate the 5-min bins without ships.

The importance of each explanatory variable was investigated using a backward stepwise model selection procedure with the *drop1* function from the R package *geeasy*^55^ to test the significance of each term, dropping non-significant (*P* > 0.05) terms and reevaluating the model until all terms were significant (Table S2-3). *P*‐value, degrees of freedom, and chi‐squared values from an ANOVA of final models were noted and marginal *R*^2^ values were calculated for each model^56^ (Table S3-4). For each variable in the final models, the average prediction of narwhal acoustic presence was visualized with 95% confidence intervals generated using a parametric bootstrap with 1000 iterations.

## Supplementary Information

Supplementary Information.

## Data Availability

The data used to support the findings of this may be made available by the corresponding author upon request.
