# Supplementary material for: Narwhal acoustic presence in Eclipse Sound, Nunavut: relationships with sea ice and responses to ships
Source: Sci Rep. 2025 Jul 2;15:23126. doi: 10.1038/s41598-025-04032-1 (PMC12223179; doi:10.1038/s41598-025-04032-1)
Supplement: Supplementary file 1 — Supplementary Information. [file 41598_2025_4032_MOESM1_ESM.pdf]

Supplemental Information

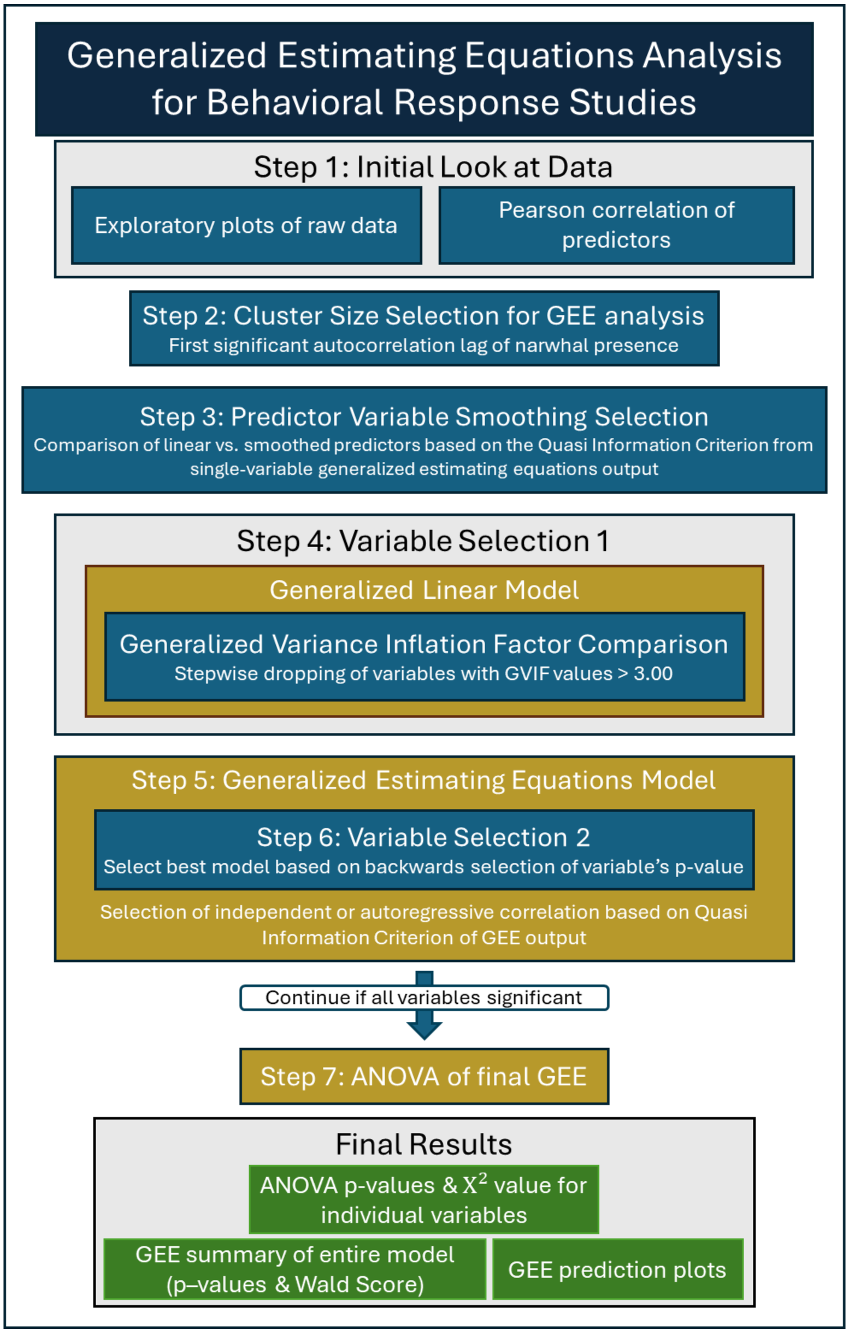

Figure S1: Flow diagram for the Generalized Estimating Equations modelling approach used in this analysis

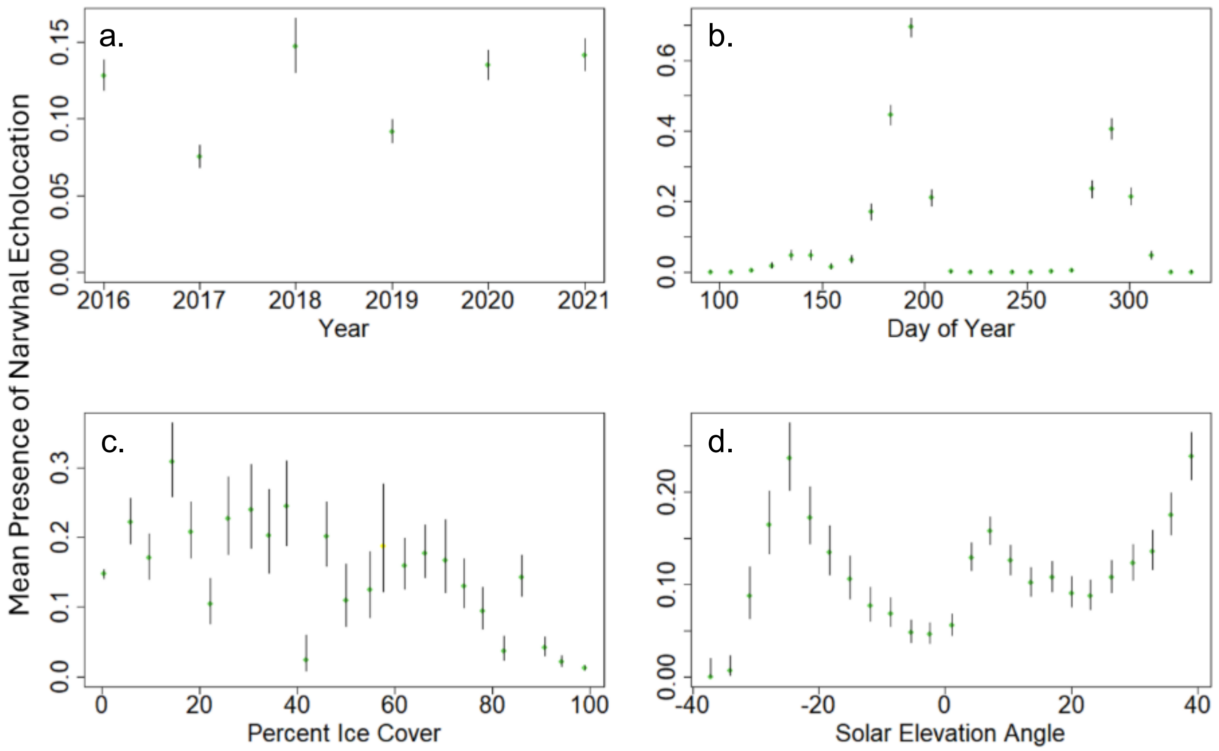

Figure S2.1: Hourly mean probability of narwhal presence between April-December 2016-2021 at site EE in relation to the explanatory variables explored in this study.

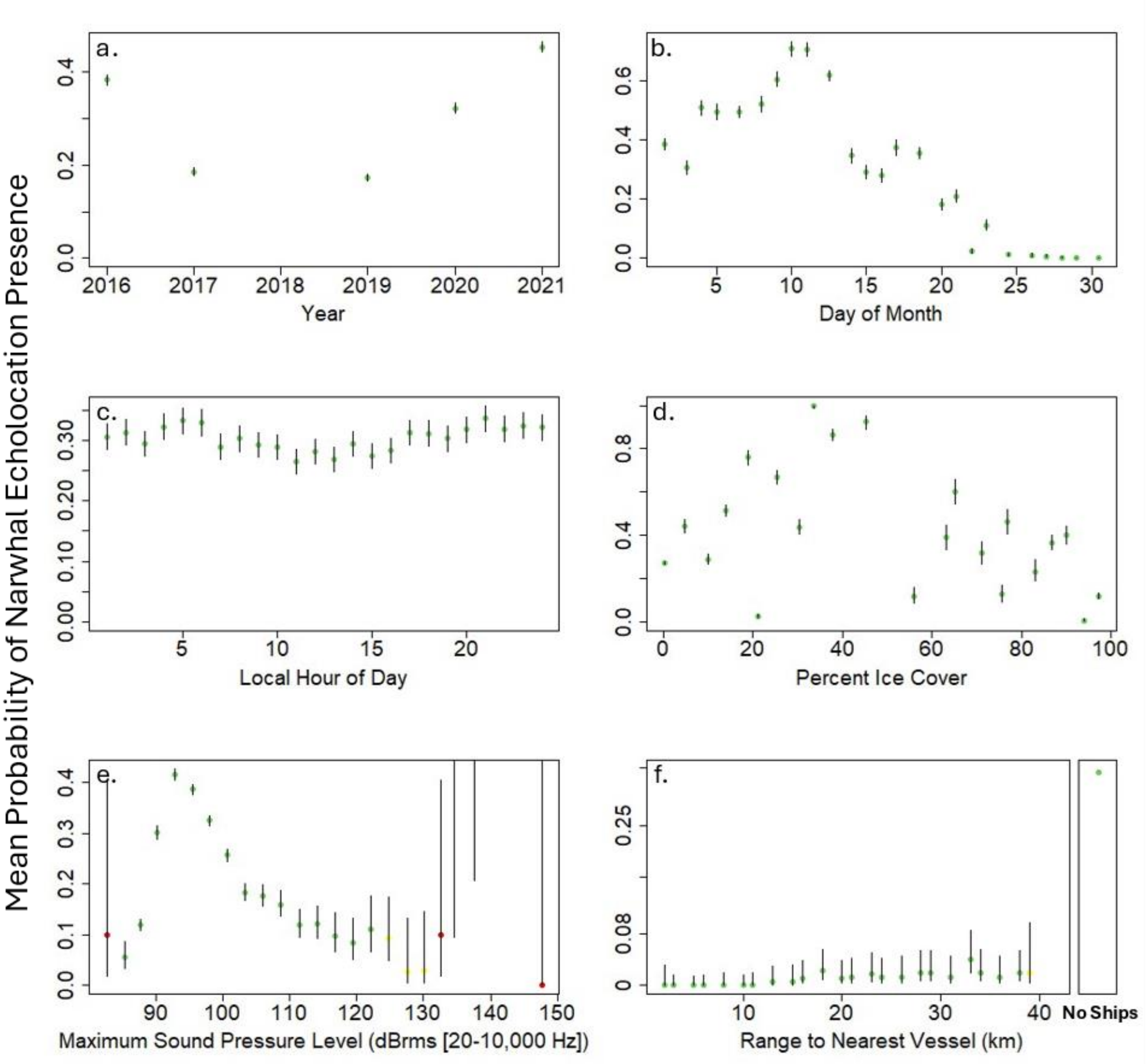

Figure S2.2: 5-minute mean probability of narwhal presence in July 2016-2021 (excluding 2018) at site EE in relation to the explanatory variables explored in this study.

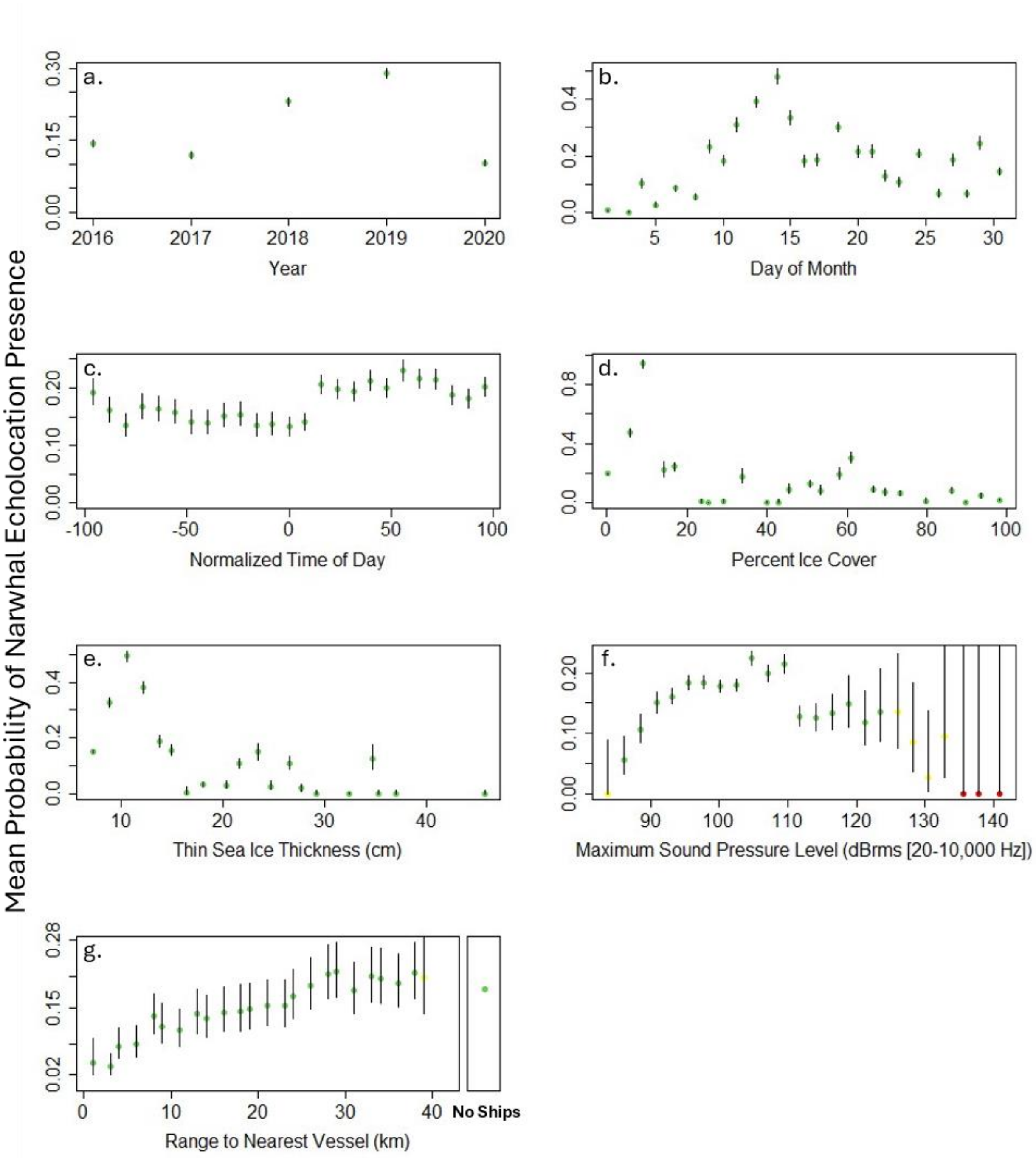

Figure S2.3: 5-Minute mean probability of narwhal presence in October 2016-2020 in at site EE in relation to the explanatory variables explored in this study.

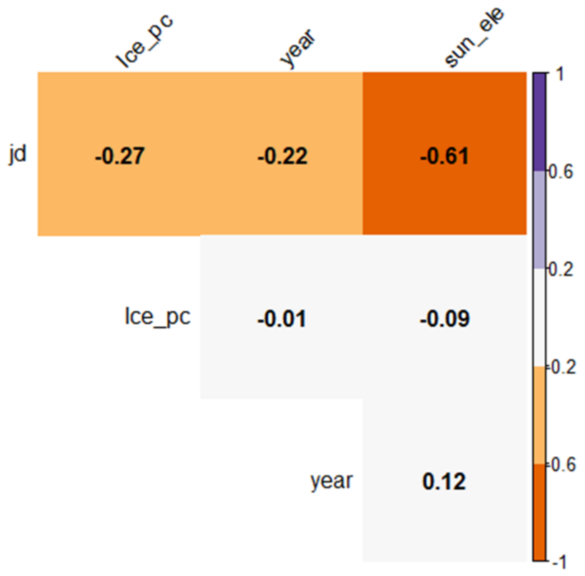

Figure S3.1: Pearson correlation coefficients of predictor variables for hourly data. As seen here, the day of year (*jd*) is highly correlated with the solar elevation angle (*sun_ele*), and shows some correlation with the year and percent ice cover (*ice_pc*).

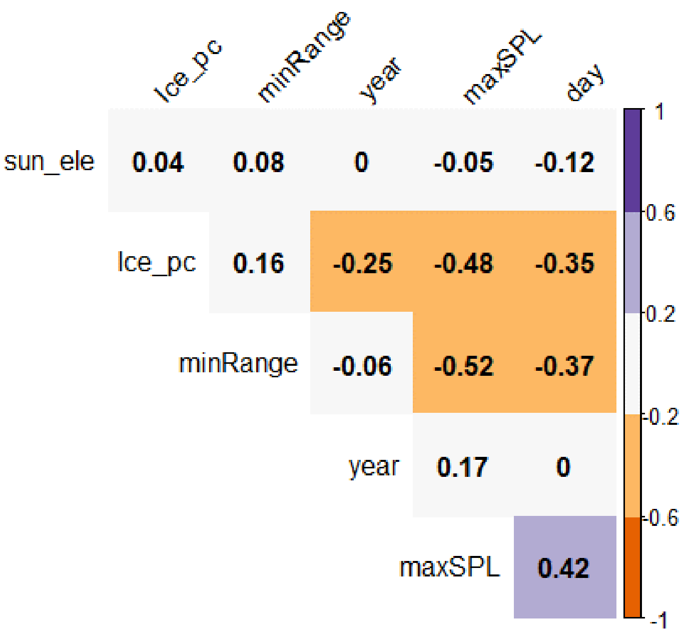

Figure S3.2: Pearson correlation coefficients of predictor variables for 5-minute binned data during the month of July. Maximum sound pressure level (*maxSPL,* dB rms [20 – 10,000 Hz]) has higher correlations with the day of month (*day*), percent ice cover (*ice_pc*), and range to nearest vessel (*minRange*).

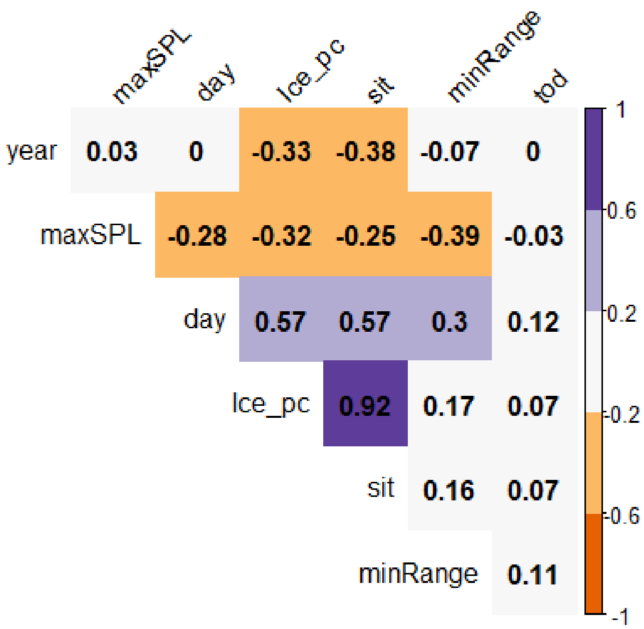

Figure S3.3: Pearson correlation coefficients of predictor variables for October 5-minute binned data. Thin sea ice thickness (*sit*) is highly correlated with percent ice cover (*ice_pc*). The day of month (*day*) also tends to have higher levels of correlation with percent ice cover and sea ice thickness.

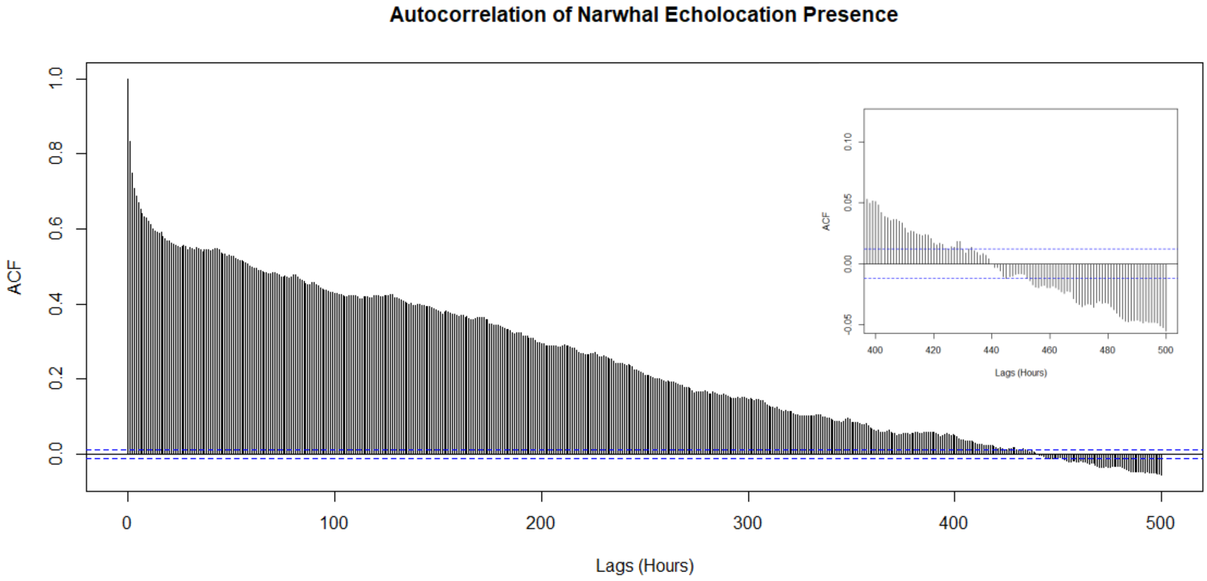

Figure S4.1: Autocorrelation function (ACF) values for each 1 hour lagged period for the annual model. Significant lag time is reached after 431 hours, approximately 18 days. This reflects the general timeframe of migration through Eclipse Sound as the periods of 0 presence in the winter and summer inflate the value of the first significant autocorrelation lag time.

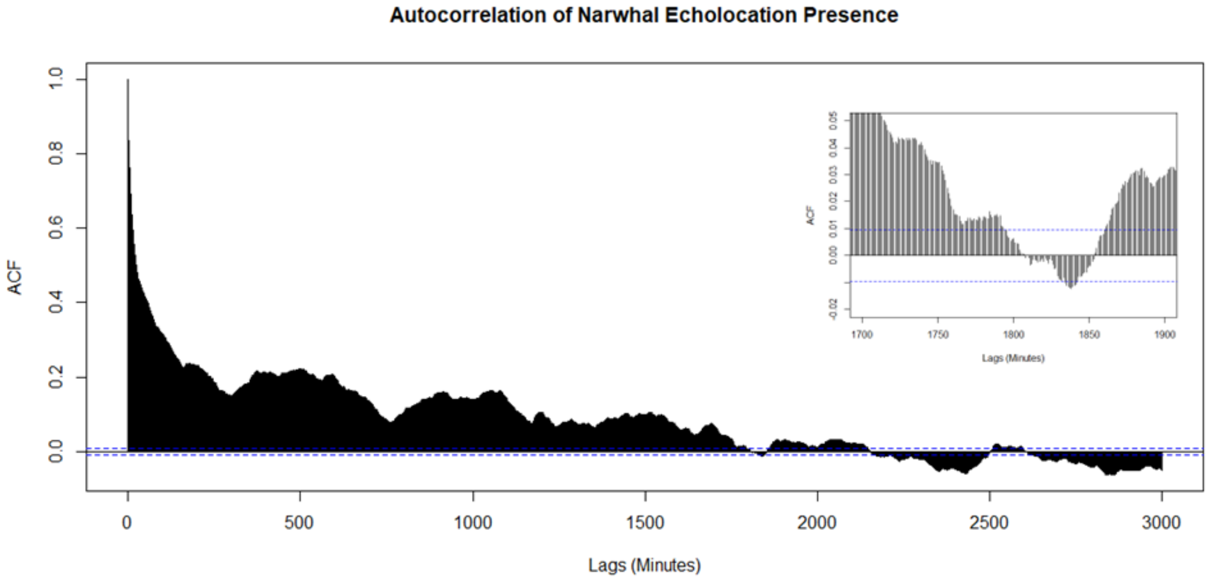

Figure S4.2: Autocorrelation function values for each five-minute lagged period for the July model. Significant lag time is reached after 2,522 five-minute periods, approximately 8.75 days. The lower significant autocorrelation lag period here compared to the annual model is due to the more constant presence of narwhals in July.

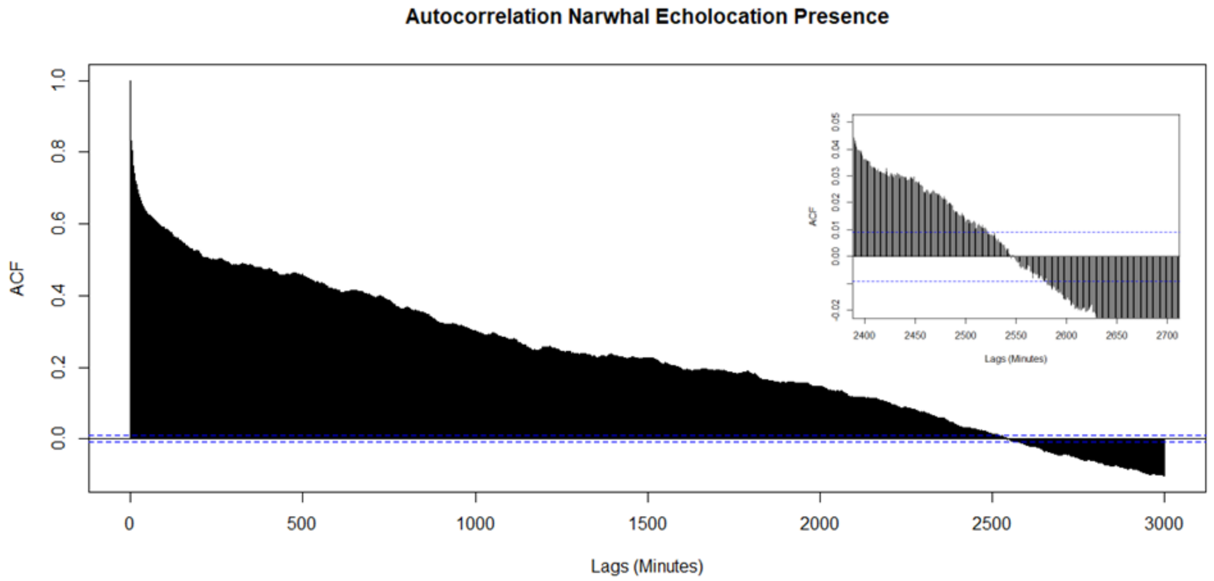

Figure S4.3: Autocorrelation function values for each five-minute lagged period for the October model. Significant lag time is reached after 1,794 five-minute periods, approximately 6.25 days. The lower significant autocorrelation lag period here compared to the annual model is due to the more constant presence of narwhals in October.

Table S1: Variable selection for annual, July, and October models. Variables were fitted to Generalized Linear Models (GLM's), and collinearity was tested with the Generalized Variance Inflation Factor (GVIF) for the selection of explanatory variables. Each subsequent test run excluded variables with GVIF > 3 (bold number values); while remaining variables were selected as a subset of explanatory variables for model building.

**Annual Model (Generic)**	GVIF Value
Variable	*Run 1*
Year	1.3
Day of Year	1.3

**Annual Model (Environmental)**	GVIF Value
Variable	*Run 1*
Solar Elevation Angle	1.1
Percent Ice Cover	1.1

**July Model**	GVIF Value
Variable	*Run 1*
Percent Ice Cover	1.0
Range to Nearest Vessel	1.0
Sun Elevation Angle	1.0

**October Model**	GVIF Value	
Variable	*Run 1*	*Run 2*
Percent Ice Cover	**16.3**	1.1
Range to Nearest Vessel	1.0	1.0
Sun Elevation Angle	1.1	1.1
**Sea Ice Thickness**	**16.2**	

Table S2: Variable selection for annual, July, and October models. Variables retained from the previous step were run through GEE's. Correlation structure was selected by the lower value under the QIC, with all models being selected for correlations structures of independence. Stepwise dropping of predictor variables from GEE's to test for individual variables significance using the drop1 function and ANOVA model, and only significant variables were retained for the final model. Variables in bold font were removed (* p < 0.05, ** p < 0.01, *** p < 0.001)

**Annual Model (Generic)**			
Correlation Structure	Variable	Wald	P-Value Pr(>Chi)
Independent	**Year**	7.302	**.199**
	Day of Year	23.134	< 0.001 ***

**Annual Model (Generic, Year removed)**				
Correlation Structure	QIC	Variable	Wald	P-Value Pr(>Chi)
Independent	12823.1	Day of Year	19.755	< 0.001 ***

**Annual Model (Environmental)**				
Correlation Structure	QIC	Variable	Wald	P-Value Pr(>Chi)
Independent	18079	Percent Ice Cover	81.347	< 0.00001 ***
		Sun Elevation Angle	24.912	< 0.0001 ***

**July Model**			
Correlation Structure	Variable	Wald	P-Value Pr(>Chi)
Independent	**Percent Ice Cover**	4.66	**0.198**
	Range to Nearest Vessel	2199.75	< 0.0001 ***
	Sun Elevation Angle	21.18	< 0.001 ***

**July Model (with Ice Removed)**				
Correlation Structure	QIC	Variable	Wald	P-Value Pr(>Chi)
Independent	53331	Range to Nearest Vessel	2587.48	< 0.0001 ***
		Sun Elevation Angle	22.56	< 0.0001 ***

October Model				
Correlation Structure	QIC	Variable	Wald	P-Value Pr(>Chi)
Independent	39237	Sea Ice Concentration	22.691	< 0.001 ***
		Sun Elevation Angle	17.510	< 0.0001 ***
		Range to Nearest Vessel	50.427	< 0.00001 ***

Table S4: ANOVA results for Final GEE models. Models were only retained if all variables were found to be significant ($p < 0.05$). Percent ice cover in the July model was removed. (* $p < 0.05$, ** $p < 0.01$, *** $p < 0.001$)

Annual Model (Generic)				
$R^2_{marginal}$	Variable	DF	χ^2	P-Value P(>|Chi|)
29.245 %	Day of Year	4	19.755	< 0.001 ***

Annual Model (Environmental)				
$R^2_{marginal}$	Variable	DF	χ^2	P-Value P(>|Chi|)
4.894%	Solar Elevation Angle	3	26.3	< 0.00001 ***
	Sea Ice Concentration	3	81.3	< 0.00001 ***

July Model				
$R^2_{marginal}$	Variable	DF	χ^2	P-Value P(>|Chi|)
5.5%	Range to Nearest Vessel	3	2587.48	< 0.00001 ***
	Sun Elevation Angle	3	36.51	< 0.00001 ***

October Model				
$R^2_{marginal}$	Variable	DF	χ^2	P-Value P(>|Chi|)
4.7 %	Sea Ice Concentration	3	18.962	< 0.001 **
	Sun Elevation Angle	3	19.095	< 0.001 ***
	Range to Nearest Vessel	3	50.427	< 0.00001 ***

GEE Model Output Summary

Table S5: Summary statistics for each GEE model with individual splines (numbered) described. (* p < 0.05, ** p < 0.01, *** p < 0.001)

Annual Model (Generic)				
Variable	Estimate	Standard Error	Wald Score	*p* - Value Pr(>|W|)
(Intercept)	4.553036	1.939174	5.512752	0.018878*
mSpline(day of year, knots = kjd, Boundary.knots = lJd, periodic = T)1	-910.156	266.0746	11.70105	0.000625***
mSpline(day of year, knots = kjd, Boundary.knots = lJd, periodic = T)2	165.7099	79.24983	4.3722	0.03653*
mSpline(day of year, knots = kjd, Boundary.knots = lJd, periodic = T)3	-2636.78	795.5421	10.98558	0.000918***
mSpline(day of year, knots = kjd, Boundary.knots = lJd, periodic = T)4	-1493.4	626.396	5.683994	0.01712*

Annual Model (Environmental)				
Variable	Estimate	Standard Error	Wald Score	*p* - Value Pr(>|W|)
(Intercept)	-1.29838	0.659206	3.879395	0.048882*
bs(solar elevation angle)1	-0.29356	1.055492	0.077355	0.780915
bs(solar elevation angle)2	-1.50518	0.884716	2.894488	0.088883
bs(solar elevation angle)3	0.436984	0.574319	0.578928	0.446733
bs(sea ice concentration)1	0.235122	1.677523	0.019645	0.888533
bs(sea ice concentration)2	1.361137	0.987909	1.898324	0.168266
bs(sea ice concentration)3	-2.54921	0.579879	19.3258	<0.00001***

July Model				
Variable	Estimate	Standard Error	Wald Score	P – Value Pr(>|W|)
(Intercept)	-11.9679	0.672913	316.3138	<0.00001***
bs(solar elevation angle)1	3.248764	0.836864	15.07045	0.000104
bs(solar elevation angle)2	-1.12836	0.374448	9.080603	0.002583
bs(solar elevation angle)3	1.532272	0.482673	10.07778	0.001501
Ship presence:bs(range to nearest ship)1	8.552247	0.333546	657.4277	<0.00001***
Ship presence:bs(range to nearest ship)2	-3.53472	0.848457	17.35607	<0.00001***
Ship presence:bs(range to nearest ship)3	10.33618	0.579743	317.87	<0.00001***

October Model				
Variable	Estimate	Standard Error	Wald Score	P – Value Pr(>|W|)
(Intercept)	-2.17421	0.59243	13.46887	0.000243***
bs(sea ice concentration)1	0.872974	1.965721	0.197224	0.656971
bs(sea ice concentration)2	-1.78119	2.273197	0.613972	0.433296

bs(sea ice concentration)3	-2.41225	0.653054	13.64417	0.000221***
bs(solar elevation angle)1	-2.12292	1.120528	3.589414	0.058149
bs(solar elevation angle)2	-0.00491	0.477357	0.000106	0.991787
bs(solar elevation angle)3	-1.91088	0.798649	5.724703	0.016728*
Ship presence:bs(range to nearest ship)1	1.633576	0.478513	11.65445	0.00064***
Ship presence:bs(range to nearest ship)2	0.075902	0.22771	0.111107	0.738888
Ship presence:bs(range to nearest ship)3	1.927693	0.501995	14.74612	0.000123***
